# An opinion on the roles of phenylalanine ammonia-lyase in the browning of fresh-cut fruit and vegetables

**DOI:** 10.3389/fnut.2025.1561620

**Published:** 2025-04-23

**Authors:** Qiu-yan Cao, Xiao Yuan, Can Zhang, Xin Deng, Yuan-yuan Jiang, Bin Wang

**Affiliations:** ^1^Guangdong Provincial Key Laboratory of Utilization and Conservation of Food and Medicinal Resources in Northern Region/College of Biology and Agriculture, Shaoguan University, Shaoguan, China; ^2^Guangdong Provincial Engineering and Technology Research Center of Special Fruit and Vegetables in Northern Region, Shaoguan University, Shaoguan, China

**Keywords:** fresh-cut fruit and vegetable, enzymatic browning, postharvest quality, phenylalanine ammonia-lyase, phenylpropanoid pathway

## 1 Introduction

Fresh-cut fruit and vegetables are becoming increasingly popular as fresh food products, valued for their convenience, health benefits, and ready-to-eat nature (1). Unlike intact produce, fresh-cut fruit and vegetables undergo various processing operations, such as peeling, slicing and dicing, prior to consumption and storage in market. Consequently, they are more susceptible to decay and discoloration, leading to a relatively short shelf life (2). Among these challenges, browning presents one of the most significant issues in the fresh-cut industry (3), resulting in an undesirable dark coloration. Such browning not only diminishes the visual appeal but also adversely impacts consumers' acceptance (4).

Furthermore, certain compounds, particularly phenolic compounds, serve as essential sources of antioxidants in fruit and vegetables. The oxidation of these phenolic compounds during the browning process inevitably results in a loss of antioxidant capacity in fresh-cut products, ultimately diminishing their associated health benefits (5). Therefore, understanding the browning mechanisms of fresh-cut produce is essential for the sustainable development of the fresh-cut industry and for producing high-quality products that meet consumers' expectations.

Over the past three decades, considerable progress has been made in understanding the browning mechanisms of fresh-cut produce, particularly concerning the roles of phenolases in the browning reaction (6). However, the specific roles of phenylalanine ammonia-lyase (PAL) in the browning of fresh-cut produce remain ambiguous, and its potential as a target for browning control strategies has not been sufficiently explored. This opinion article aims to delve into the nuanced roles of PAL in enzymatic browning, emphasizing its potential implications for regulating the browning development of fresh-cut fruit and vegetables.

## 2 Current perspectives on the browning mechanisms of fresh-cut produce

It is widely recognized that food browning can be categorized into two distinct types: enzymatic and non-enzymatic browning (7). The latter occurs predominantly during the heating of food and is exemplified by processes such as the Maillard reaction and caramelization. In contrast, enzymatic reactions are the primary contributors to browning in fresh-cut fruit and vegetables (8).

Fresh-cut produce experiences extensive mechanical injury, which compromises the integrity of the cell membrane and leads to the leakage of cellular contents and exposure to oxygen (9, 10). This enzymatic browning process is initiated when phenol-metabolizing enzymes, such as polyphenol oxidase (PPO) and peroxidase (POD), come into direct contact with phenolic compounds in the presence of oxygen, resulting in phenolic oxidation and the subsequent formation of brown pigments (11, 12). Accordingly, current strategies employed for controlling browning in fresh-cut products focus primarily on regulating PPO activity.

However, some studies have indicated that PPO or POD activity are not always directly correlated with the browning rate in fresh-cut products. For instance, a study of five potato cultivars (Agria, Cara, Liseta, Monalisa, and Spunta) revealed varying susceptibility to browning, yet there was no significant correlation between the rate or degree of browning and the activities of PPO and POD in fresh-cut potatoes (13). In another study involving fresh-cut Chinese water chestnuts, treatments with both exogenous ascorbic acid (AsA) and ferulic acid (FA) were found to effectively inhibit the activities of PPO and POD during storage. However, only FA treatment was effective in reducing browning, while AsA treatment did not show a significant effect (14). In comparison to normal mango fruit, the total phenolic concentration in under-skin browning mango exhibited a 7.4% increase, while the activities of PPO and POD decreased by 19.0% (15). These researches together suggest that, in addition to PPO or POD, other important factors also have significant roles in the browning of fresh-cut produce.

## 3 PAL plays key roles in the browning of fresh-cut products

### 3.1 Wounding induces PAL protein and gene expression

Fresh-cut processing, often referred to as minimal processing, represents a form of wounding stress that induces mechanical injury to the intact fruit or vegetable (16). In response to wounding stress, plants activate a range of defense mechanisms designed to mitigate the effects of injury. Among the various strategies employed by plants, the phenylpropanoid pathway plays a crucial role in synthesizing protective compounds in response to wounding stress. This pathway is responsible for the production of a diverse array of secondary metabolites, including flavonoids and other phenolic compounds (17). Phenolic compounds and flavonoids in wounded fresh-cut fruit and vegetables are produced rapidly, partly to facilitate lignin synthesis as a response to wounding stress (16).

PAL is the first enzyme in the phenylpropanoid pathway, responsible for converting L-phenylalanine into a range of phenolic compounds, such as phenols, phenolic acids, anthocyanins, flavonoids, isoflavones, and other polyphenolic metabolites in plants (18, 19). It is well documented that wounding stress induces PAL activity and gene expression in fresh-cut products (16). Studies had shown that the expression levels of four *PAL* genes, as well as PAL activity, were significantly elevated in fresh-cut lettuce stems following wounding (20). In fresh-cut taro, PAL activities and the expression of three *PAL* genes increased at 3 d compared to 0 d, indicating that slicing induces *PALs* expression in fresh-cut taro (21).

During the browning process of fresh-cut peaches, the activities of PAL, cinnamate-4-hydroxylase (C4H), and 4-coumarate: CoA ligase (4CL), two key enzymes in the phenylpropanoid pathway, were found to increase with the extent of browning, while hydrogen sulfide (H_2_S) treatment significantly inhibited both browning and these enzyme activities (22). Similar results regarding the induction of PAL through minimal processing have been observed in fresh-cut apples (23), lettuce stems (24), potatoes (25) as well as sweet peppers (26). Collectively, these studies suggest that wounding induces PAL activity and gene expression, which correlate positively with the development of browning, illustrating the positive roles of increased PAL activity in the browning of fresh-cut products.

### 3.2 PAL directly contributes to browning by promoting flavonoid biosynthesis

As the above mentioned, PAL is a key enzyme in the biosynthesis of various phenolics through the phenylpropanoid pathway. Many intermediate products in this pathway exhibit deep coloration, particularly flavonoids (27). Flavonoids represent one of the three major classes of plant pigments and encompass six primary subgroups, such as chalcones, flavonols, anthocyanins, and proanthocyanidins (28). The accumulation of these pigmented compounds results in a browning or yellowing appearance. For instance, the accumulation of quercetin 3-O-glycosides, the predominant flavonols in apple fruit, results in a bright yellow to yellow hue that varies with concentration (29). This browning symptom, resulting from the accumulation of flavonoids, differs from the products traditionally attributed to PPO activity, which are primarily formed through the oxidation of phenolic compounds.

PAL activity has been linked to the concentrations of flavonoids and other phenolic compounds in plants. *StlA*, a gene encoding PAL from *Photorhabdus luminescens*, has been shown to involve in the production of stilbene antibiotic (30). Treatments with UV-B and blue light significantly promoted the accumulation of quercetin 3-O-glycosides, including quercetin 3-O-glucoside, quercetin 3-O-galactoside, and kaempferol 3-O-galactoside, through the phenylpropanoid pathway by inducing PAL activity (29). In cotton, the expression levels of key genes including *PAL* involved in flavonoid biosynthesis were significantly higher in brown fibers compared to white and green fibers. The increased expression contributed to elevated levels of total flavonoids and proanthocyanidins, resulting in the brown coloration of cotton fibers (28).

Wounding-induced PAL may play a significant role in the accumulation of flavonoids and the onset of browning. Eriodictyol and naringenin are two flavonoids derived from the phenylpropanoid pathway (31). Peeling induced PAL and other key components in this pathway, resulting in a positive correlation between the browning of fresh-cut Chinese water chestnuts and increased levels of eriodictyol and naringenin (14). In apple fruit, peeling and cutting stimulated the accumulation of several flavonoids, including epicatechin, catechin, hyperin, quercitrin, and rutin, which accompanied by increased PAL activity and gene expression (32). These studies again confirm that PAL plays a crucial role in the biosynthesis of colored compounds through the phenylpropanoid pathway.

### 3.3 PAL may lead to an increase in phenolic substrates available for oxidation

Enzymatic browning mediated by PPO is a widely recognized mechanism responsible for the discoloration of fresh-cut products (33, 34). According to this theory, brown pigments and melanin are formed under the catalysis of PPO in plants. However, PPO has a broad range of substrates in higher plants (35). As mentioned earlier, increases in PAL activity lead to a corresponding rise in the concentration of various phenolic compounds. For instance, elevated PAL activity contributes to the accumulation of cinnamic acid, caffeic acid, chlorogenic acid, hydroxybenzoic acid, catechin, and sinapic acid, in fresh-cut broccoli (36). In cold-stressed fresh walnuts, increases in the levels of vanillic acid, 4-hydroxybenzoic acid, syringic acid, and 2,4-dihydroxybenzoic acid, were consistent with increased PAL activity (37).

Furthermore, the induction of PAL also leads to the accumulation of various precursors involved in phenolic biosynthesis, with this enzyme regulating the flux of these precursors into the phenolic network (38). For instance, *p*-Coumaroyl-CoA serves as a crucial precursor for flavonoid metabolism and other phenolic pathways, and increased PAL activity results in greater accumulation of *p*-Coumaroyl-CoA in broccoli (39). In this context, PAL induction following fresh-cut processing facilitates *de novo* biosynthesis or accumulation of phenolics, providing essential substrates for oxidative reactions mediated by PPO or POD.

## 4 The inactivation of PAL activity could restrict the browning development

The key roles of PAL in the browning of fresh-cut produce are further reinforced by observations that inactivating PAL activity can effectively limit browning in these products. The application of PAL inhibitors, such as α-aminooxyacetic acid, 2-aminoindan-2-phosphonic acid, and α-aminooxy-β-phenylpropionic acid, has been shown to significantly inhibit browning in fresh-cut lettuce (40). In another study focused on fresh-cut lettuce, researchers found that treating lettuce stems with acetic acid suppressed butt discoloration by inhibiting PAL both enzymatically and transcriptionally (20). Recent studies have also reported similar beneficial effects resulting from the inactivation of PAL activity in other produce, including fresh-cut taro (41), potatoes (42), Chinese water chestnut (43), and pineapple (44). By demonstrating that PAL inactivation can effectively mitigate browning, these researches pave the way for new strategies to maintain the visual quality and extend the shelf life of fresh-cut produce.

## 5 Conclusion and outlook

**Figure 1:**
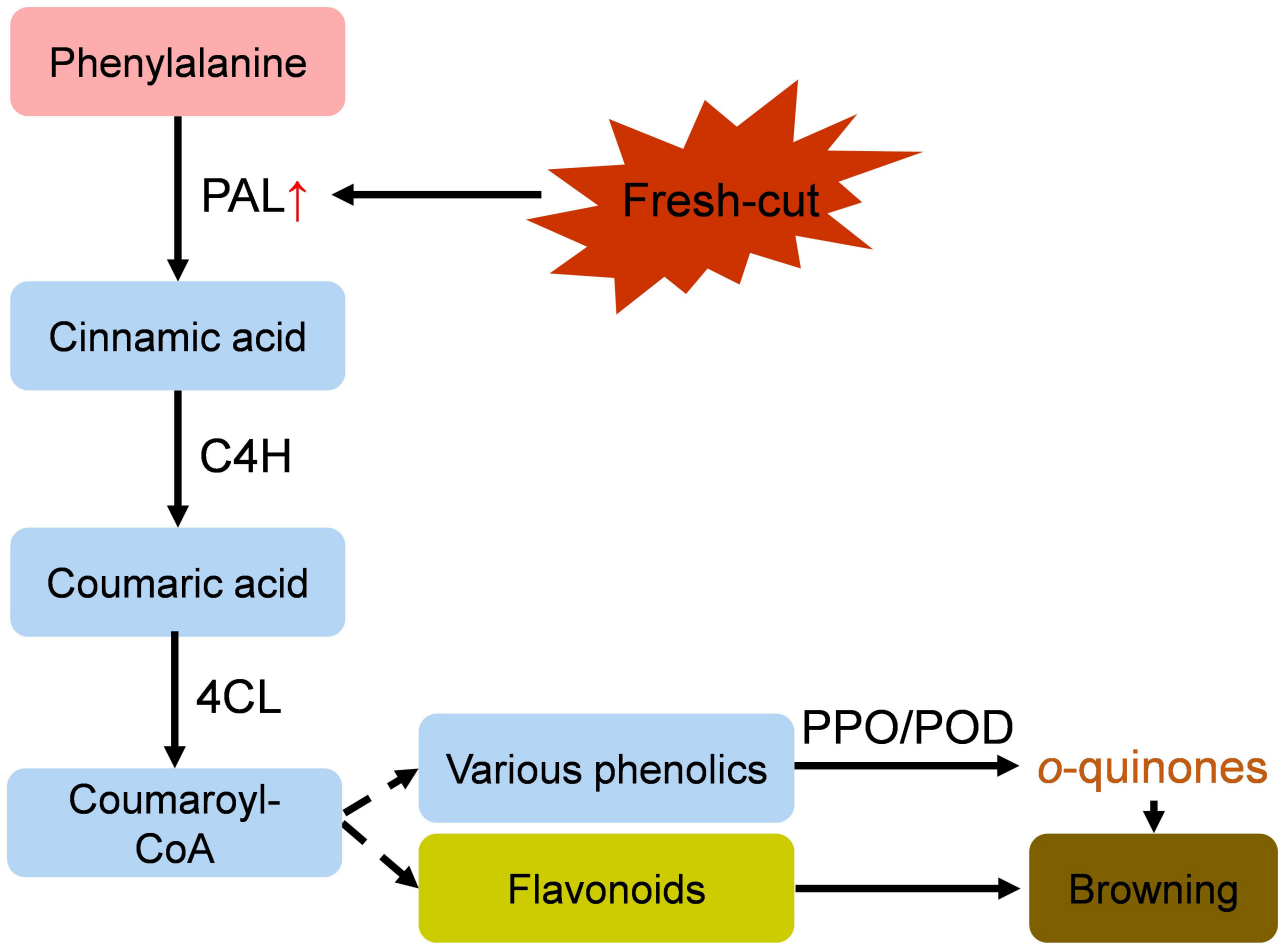
A working model illustrating the centrality of PAL in regulating enzymatic browning of fresh-cut produce. The upward arrow (↑) indicates promotive or inducible effects.

Up to now, nearly all existing studies have focused on the important roles of PPO and/or POD in enzymatic browning of fresh-cut fruit and vegetables, while the roles of PAL in product browning has received limited attention. This opinion summarizes the multifaceted roles of PAL in the browning of fresh-cut produce, highlighting that PAL may be a key factor in regulating browning reactions. In a word, fresh-cut operations induce PAL at both the gene and protein levels. Consequently, the increased activity of PAL contributes to browning by either inducing the accumulation of pigmented compounds through the phenylpropanoid pathway or by providing sufficient phenolic substrates for PPO-mediated oxidation. Figure 1 illustrates the critical roles of PAL in the browning process of fresh-cut produce.

In the future, more studies should be conducted to further explore the detailed mechanisms by which PAL contributes to browning, as well as its actual color contribution to fresh-cut produce. For this purpose, novel analytical methods need to be developed with the aim of controlling browning reactions in fresh-cut fruit and vegetables from a PAL perspective. A specific example is the exploration of the potential applications of PAL inhibitors. Additionally, it is essential to identify key genes that encode PAL protein and regulate browning in fruit and vegetables. These works are crucial for developing novel varieties that are fully resistant to browning through genetic engineering or transgenic technologies.

Overall, this work contributes to a better understanding of PAL's roles in the browning of fresh-cut food and points out directions for developing more effective browning control methods through modulating PAL activity.
